# GUDM: Automatic Generation of Unified Datasets for Learning and Reasoning in Healthcare

**DOI:** 10.3390/s150715772

**Published:** 2015-07-02

**Authors:** Rahman Ali, Muhammad Hameed Siddiqi, Muhammad Idris, Taqdir Ali, Shujaat Hussain, Eui-Nam Huh, Byeong Ho Kang, Sungyoung Lee

**Affiliations:** 1 Department of Computer Engineering, Kyung Hee University, Seocheon-dong, Giheung-gu Yongin-si, Gyeonggi-do 446-701, Korea; E-Mails: rahmanali@oslab.khu.ac.kr (R.A.); siddiqi@oslab.khu.ac.kr (M.H.S.); idris@oslab.khu.ac.kr (M.I.); taqdir.ali@oslab.khu.ac.kr (T.A.); shujaat.hussain@oslab.khu.ac.kr (S.H.); johnhuh@khu.ac.kr (E-N.H.); 2 Department of Computing and Information Systems, University of Tasmania, Hobart Tasmania 7005, Australia; E-Mail: byeong.kang@utas.edu.au

**Keywords:** unified dataset, data fusion, data model, rough set theory, knowledge acquisition, reasoning, clinical trials, social media, sensors

## Abstract

A wide array of biomedical data are generated and made available to healthcare experts. However, due to the diverse nature of data, it is difficult to predict outcomes from it. It is therefore necessary to combine these diverse data sources into a single unified dataset. This paper proposes a global unified data model (GUDM) to provide a global unified data structure for all data sources and generate a unified dataset by a “data modeler” tool. The proposed tool implements user-centric priority based approach which can easily resolve the problems of unified data modeling and overlapping attributes across multiple datasets. The tool is illustrated using sample diabetes mellitus data. The diverse data sources to generate the unified dataset for diabetes mellitus include clinical trial information, a social media interaction dataset and physical activity data collected using different sensors. To realize the significance of the unified dataset, we adopted a well-known rough set theory based rules creation process to create rules from the unified dataset. The evaluation of the tool on six different sets of locally created diverse datasets shows that the tool, on average, reduces 94.1% time efforts of the experts and knowledge engineer while creating unified datasets.

## 1. Introduction

Generally, a successful decision support system relies on high quality information created either by a knowledge engineer or automatically generated from the data. The first approach is expensive while the other is constrained by the availability and quality of data. A huge volume of human-centric personal data is available but integrating them from various sources into a unified dataset is challenging. The integration of multiple heterogeneous data sources is an important research issue that is not limited to the healthcare arena. To enable the use of healthcare data in clinical decisions, automatic generation of a single unified dataset is desirable [1]. This task is very challenging due to a number of technical issues, such as semantic heterogeneity, different naming conventions, resolving attributes’ values conflicts, finding intrinsic relationships, handling missing values and overlapping information and converting local datasets into global unified data model [2,3]. This paper focuses on the last four challenges and leaves the rest as future work.

Existing biomedical application tools are generally based on data from a single data source, specifically, the clinical trials and observations [4,5,6,7]. However, the success of a medical practitioner depends upon the use of data from disparate data sources to diagnose and predict results. Medical practitioners are not expert at using disparate types of data for analysis and prediction. To overcome this problem, automatic software tools are needed to merge biomedical data into a unified dataset. The purpose of this study is to develop software tool to solve this problem.

**Figure 1:**
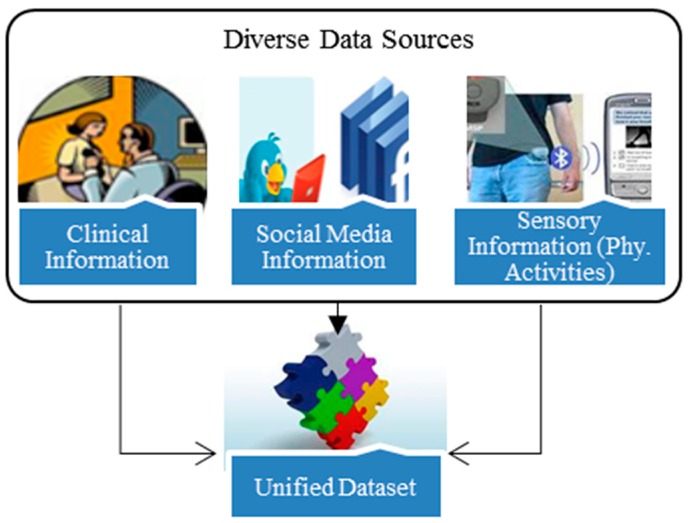
Scenario to integrate diverse datasets into a unified dataset.

At the Ubiquitous Computing Laboratory, Kyung Hee University, we are working on the development of a cloud-based clinical decision support system (*CDSS*) for chronic disease patients [8]. This system is supposed to predict diabetes type (*i.e*., type 1 or type 2 or no diabetes) in patients and generate recommendations. The proposed *CDSS* takes data from multiple data sources, such as sensors, user profiles, social media and clinical trials, as shown in Figure 1.

This system is intended to generate clinical and personalized wellness recommendations using virtual medical record (vMR)-based and machine learning-based reasoning methodologies. Technically, vMR-based reasoning is internally a vMR-enabled Medical Logic Module (MLM)-based reasoning methodology that is used for clinical recommendations. MLM encapsulates medical logic for corresponding domain and a set of logically related MLMs represents knowledge base. MLM uses HL7 vMR data model to enable integration of knowledge base with external healthcare system. This reasoning approach has been implemented and clinical recommendations are being generated [8]. The machine learning-based reasoning methodology is under development and is supposed to use different machine learning (ML) and rough set theory-based algorithms to first create knowledge and then use for personalized recommendations. Here, the use of these methods requires a highly abstracted preprocessed unified dataset, which has data combined from all relevant data sources (Figure 1). However, generation of a unified dataset with an automatic software tool is a challenging problem. A dataset generated from diverse data sources enables better analysis and decision making as compared to a dataset from a single source.

The main research issues that need to be resolved are seamless global unified data modeling, smartly tackling overlapping attributes, efficient fusion of heterogeneous datasets and designing an easy-to-use graphical interface. To resolve these issues, we propose a linear sequential data modeling approach with an expert-centric priority-based approach to integrate data sources into a unified dataset. We have solved these issues in our proposed GUDM and the associated “data modeler” tool. The main contributions of the work include:

(1) Automatically defines a global unified data model at run time from diverse data sources seamlessly.
(2) Resolves the problem of overlapping attributes across multiple datasets by utilizing the proposed expert-centric priority based approach.
(3) Produces the unified dataset in a consistent way and makes it easy for automatic learning and reasoning systems/tools to efficiently acquire knowledge from it that can be used for different types of reasoning and prediction services.

Other auxiliary contributions include: (a) a domain independent solution, *i.e*., the “data modeler” can be used in domains other than the medical domain; (b) provides a detailed view of data to a knowledge engineer to easily address any potential problem; (c) helps knowledge engineers to import and export datasets in multiple formats to increase the usability of the system; (d) offers the possibility of future extension due to the flexible framework of the system.

Apart from the above mentioned issues, privacy is also one of the key concerns of a cloud-based clinical recommendation system due to patient’s personal data. Though privacy is not focus of this study, however for reader’s interests we add information on cloud-based privacy. Hussain *et al*. [8] has discussed the idea of data anonymization in his study of CDSS for diabetes recommendation to keep the diabetes patient’s data private on the cloud. Moreover, in our lab, cloud-based privacy research is an ongoing work and oblivious user management techniques have been proposed [9,10] for keeping user’s personal data intact on public cloud.

We have simulated the “data modeler” with a brief scenario of diabetes datasets. The current version (version 1.0) of application can be downloaded from the *SourceForge* [11].

The rest of the paper is organized as follows: Section 2 describes the related work and Section 3 presents the GUDM model and architecture of the associated “data modeler” tool. Section 4 focuses on generating a unified dataset with an expert-centric priority-based approach. Section 5 discusses the proposed “data modeler” tool and Section 6 presents a case study scenario and its workflow on the “data modeler”. Section 7 evaluates the proposed tool while Section 8 discusses the significance, challenges and limitations of the proposed system. Section 8 concludes the work with future directions.

## 2. Related Work

The integration of multiple heterogeneous data sources into a single dataset is an active area of research. The motivation for this work is to enable domain experts to make highly accurate decisions. In literature, a huge amount of work can be found on the same topic covering different domains [12]. In domains such as business [13,14], engineering [15] and data mining [16], people have paid great attention to the issue. In the medical domain, *SAS* clinical data integration [17] transforms clinical data into standard *SDTM* and *CRT*-*DDS* models for analysis. The MotifLab tool [18] provides a flexible framework which allows users to easily incorporate different kinds of information from genome datasets for detailed analysis and visualization. This tool enables users to obtain relevant data from genome datasets and use it in combination with existing motif discovery tools in different ways to perform analysis. Similarly, Anduril [19] is a framework used for the integration and analysis of heterogeneous data and generates a summary report for it. They use a website to show the most relevant features of each gene simultaneously. This system processes large scale datasets and integrates knowledge from bio-databases. Likewise, to analyze the health conditions of children, an integrated data model (called *HeC*) has been proposed in the literature [1] which standardizes the heterogeneous biomedical information of children. This model is linked to medical ontologies, which provide database query services to the clinicians for accessing the required data. The structure of *HeC* integrated data model consists of three components: data, metadata and semantics. First, they model the domain conceptually and then define the three components mentioned. A Kernel-based statistical learning algorithm has also been used in literature [20] to combine heterogeneous genome-wide datasets. This approach is evaluated by classifying yeast ribosomal and membrane protein data. An unsupervised feature selection method, proposed in the literature [21] is applied in bio-informatical cancer research. The selection method selects relevant features from messenger *RNA* and *miRNA* (*i.e*., two data sources) and maps them into a single target dataset. The geometrical structure of the target dataset is adopted from the basic relationship among instances and the geometry-dependent covariance of the heterogeneous data sources. In the same way, a multi-kernel learning (*MKL*) approach [22] is applied to a single *MRI* data set, demographic and genetic information for the prediction of Alzheimer disease. In medical environments, where the clinical data is distributed across several sites, integration approaches have also been tried [23,24,25,26]. Apart from these techniques, Bayesian and matrix factorization-based approaches for fusion can also be found in the literature [27,28]. Similarly, the process of integration is not only restricted to non-imaging datasets, but has also been used for biomedical imaging data, as discussed in the literature [12,29,30,31,32,33,34,35,36,37]. Prioritization based approaches discussed in [38,39] have also been used in the literature. They help clinicians to quickly determine and access the required level of visual information of a particular brain cancer case from the brain MRI. The approach they have proposed is focused on the organization and prioritization of medical imaging data for further examination.

In the literature discussed so far, the proposed approaches assume that the required data is already available in a structured and uniform format which is ready to be used by the learning systems for decision making. Similarly, in some of the approaches, they have defined a single structured schema and expect to fetch data into this schema from the diverse schemas of the individual data sources to form the integrated dataset. However, in reality, data resides in diverse datasets with different schemas. Apart from these, in the literature, there is very limited attention paid to the concept of early integration of multiple non-imaging datasets, which is an important issue for generating a consistent unified dataset. To overcome these issues, we are motivated by the idea of early integration (rather than late and intermediate integrations) of diverse data sources into a unified dataset by exploiting the proposed concept of user-centric priority-based data fusion. The rationale behind early integration in combination with user-centric priority-based approach is that the inconsistencies and anomalies in the individual datasets are resolved before data is combined in a unified dataset.

## 3. Global Unified Data Model

The problem of automatic generation of a unified dataset as considered in this study can be informally defined as follows: 

Given a set of diverse datasets, a set of preprocessing and unified data modeling methods are used to generate a unified dataset that can be used to create rules/knowledge to facilitate reasoning and prediction services.

**Figure 2:**
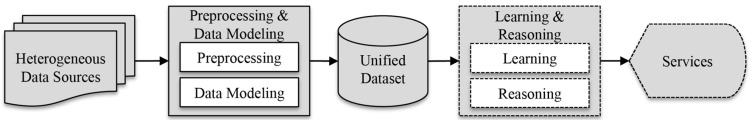
Global unified data model for generating unified dataset.

Now, we formally define the problem of automatic generation of a unified dataset and introduce notation that we will be using throughout this paper. Let *DS* denote a set of diverse datasets with *PM* as the preprocessing methods and *DM* as the data modeling methods to create a unified dataset *UD* that can be learned. *LR* represents algorithms/tools for providing various types of reasoning and prediction services. Abstractly, the proposed model (shown in Figure 2) and its working scenario can be represented as a 5-tuple: *< DS*, *PM*, *DM*, *UD*, *LR >*, where:

- *DS* (*Diverse data sources*): the set of diverse datasets recording different information on the same individuals.
- *PM* (*Preprocessing methods*): a set of methods used for loading *DS*, finding missing values, finding intrinsic relationships across multiple datasets *DS* and making the datasets uniform using reduction technique.
- *DM* (*Data modeling methods*): the set of methods used to prioritize the datasets, define a unified structure for the data model and log data from the diverse *DS.*
- *UD* (*Unified dataset*): the dataset created from *DS* by exploiting *PM* and *DM.*
- *LR* (*Learning and reasoning*): the set of machine learning algorithms/tools used for learning the data stored in *UD* to enable reasoning and prediction services for new problems (this is beyond the scope of this study and will be considered in future work).

**Figure 3:**
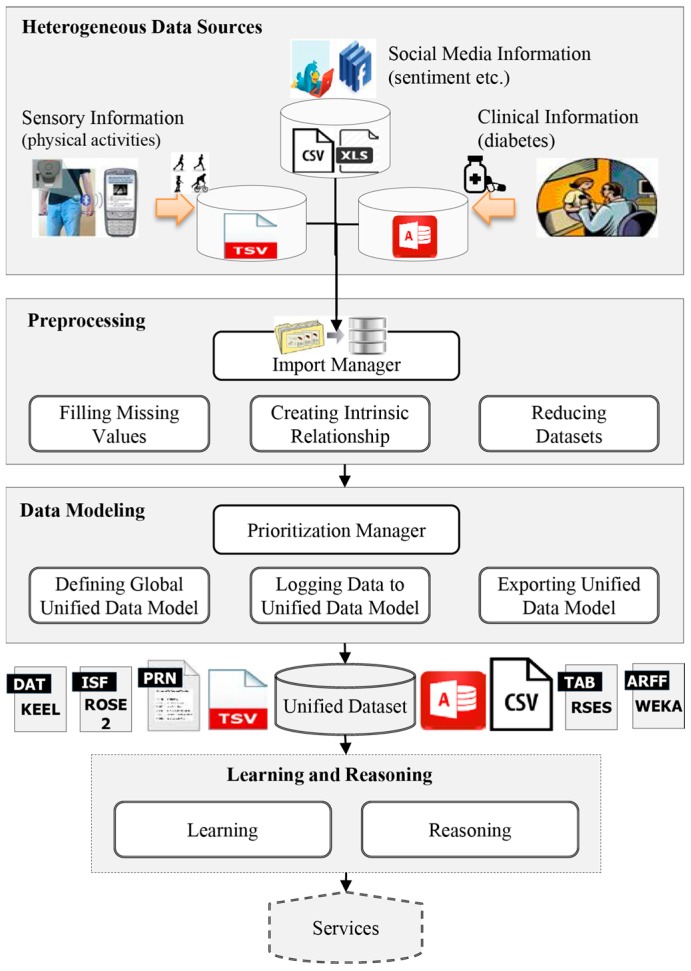
Architecture of the “data modeler” based on global unified data model.

Each item of the proposed model has a separate module is pictorially represented (Figure 3) by the architecture of the “data modeler” tool. These modules are designed according to a workflow mechanism which follows a series of sequential processing steps. Each step in the workflow executes a specific task in the generation of a unified dataset.

The first module of the “data modeler” tool describes external diverse data sources *DS* which need to be combined in the unified dataset. There can be several source files in the *DS* which need to be transferred to the required input file formats (*i.e*., .csv, .xls, .tsv and .accdb) of the tool. In our diabetes scenario (Section 6), these data sources include clinical observations, social media information and sensory information (physical activities).

The second module, *PM*, includes a set of preprocessing methods that are necessary for essential tasks, such as importing the datasets, finding intrinsic relationships, handling missing values, and making the datasets uniform (using the reduction process). The number and types of the datasets to be imported are based on the user’s requirements, *i.e*., an arbitrary number of datasets can be imported to the “data modeler”.

The third module, denoted by *DM* is a key module of the “data modeler” which includes prioritization of datasets, defining a global unified data model, resolving overlapping attributes, logging data into a unified model and exporting the dataset. The prioritization manager is responsible for assigning priorities to the preprocessed datasets according to their credibility. The credibility of a dataset is assigned based on the domain expert’s feedback. The priority values help to resolve the problems of handling overlapping features while combining the datasets. The output of the data modeling module is the unified dataset (*UD*)*.*

The last module of the “data modeler” is learning and reasoning (*LR*) that takes the *UD* and uses it in commonly used machine learning-based data analysis and knowledge discovery tools, such as *WAKA* [40], *KEEL* [41], *ROSE* [42], *RSES* [43] *etc*. for acquiring knowledge. This knowledge can be used for different types of reasoning and prediction services. In Figure 3, this is denoted by dotted lines.

## 4. Methods: Generation of a Unified Dataset

For the automatic combination of multiple data sources into a unified dataset, we propose an expert-centric priority-based approach. According to this approach, priorities are assigned to different datasets based on the credibility of their data sources. The priority levels are defined by domain experts and are helpful during the combination process to solve a number of problems. The whole process of unified dataset creation, implemented in a “data modeler” tool is divided into different phases described in subsequent sections.

### 4.1. Heterogeneous Data Sources

To understand the whole process, consider a set of “*n*” heterogeneous datasets denoted by $DS=\left\{ds_{1},ds_{2},\;\ldots ,\;ds_{n}\right\}$, e.g., {clinical, social media, sensory, *etc*.}, where each $ds_{i}$ is characterized by m attributes, *i.e*., A$\;=\left\{a_{1},a_{2},\;\ldots ,\;a_{m}\right\}$. To explain the process, a running example of the following three datasets is taken into account. A detailed description of these datasets is given in Section 5.

*ClinicalDataset =* {*ID*, *Gender*, *TC*, *TG*, *LDL*, *HDL*, *SGOT*, *SGPT*, *FBS*, *SBP*, *DBP*, *Weight*, *Height*, *Hypoglycemia*, *HbA1c*, *diabetesType*}
*SocialmediaDataset =* {*ID*, *Gender*, *Symptoms*, *Sentiments*, *Activities*}
*SensoryDataset =* {*ID*, *Activities*}

### 4.2. Preprocessing Phase

The preprocessing phase is one of the key features of any knowledge discovery system. Therefore we perform some of the most commonly used pre-processing tasks that are essential for a credible unified dataset. These include missing value completion, finding intrinsic relationships among all imported datasets and making the datasets uniform. Apart from all these tasks, assuring quality of healthcare data is an important aspect of preprocessing that includes accuracy, completeness, consistency, relevance, timeliness and usability for high quality knowledge creation and decisions [44]. However, we only focus on the methods that are described in detail below.

#### 4.2.1. Import Phase

In the first phase, a knowledge engineer imports heterogeneous datasets to the working memory of a “data modeler” tool. The compatible input formats are comma separated value (.csv), excel file format (.xls), tab-separated (.tsv) text files and MS Access database files (.accdb). A knowledge engineer can import an arbitrary number of datasets to the “data modeler” environment based on his/her requirement. If DS denotes the set of n available datasets that can be imported to the system, then the process can be represented as in Equation (1):

(1) $$DS=\overset{n}{\underset{i=1}{\sum }}import\left(ds_{i}\right)$$

where the import function takes original datasets as arguments from the computer as a browse option and imports them into the “data modeler” working memory for further processing. In this case the output will be: *DS =* {*clinicalDataset*, *socialMediaDataset*, *sensoryDataset*}.

#### 4.2.2. Filling Missing Values

A dataset with missing values may not preferably be used for knowledge acquisition. Therefore we adopt the most commonly used technique for missing values, which was originally proposed by Grzymala-Busse [45]. According to this method, the missing values are replaced by the most common value and the mean value for nominal and numeric types of attributes, respectively. If DS′ is used to denote the set of all datasets after applying the method for filling in missing values, then the process can be represented as follows:

(2) $$DS\prime =\overset{n}{\underset{i=1}{\sum }}hFill\left(ds_{i}\right)$$

In Equation (2), the function hFill takes all the imported datasets sequentially as arguments and fills the holes using the adopted method. In this case, the holes are represented by empty spaces or the keywords NULL or MISSING (case insensitive). The filled datasets are either written back into the same datasets or into new datasets denoted by DS′. The process of completing the missing values can also be left to a machine learning algorithm, once the unified dataset is created. However, the idea of filling it at the early stage is to resolve this problem locally within its own dataset rather than propagating it to the unified dataset. Filling missing values before combining the datasets is more reliable than applying it in the later stage when fusion is done.

#### 4.2.3. Finding Intrinsic Relationships

One of the main issues faced when combining multiple datasets is the structure in which the instances appear in datasets. If an instance with ID equal to 1 (representing the record of subject 1) appears at position 10 in dataset 2 (representing the record of the same subject 1) then straightforward combination does not work. This mismatch of instances of the datasets across all or some of the datasets is termed non-intrinsic and should be overcome before the actual combination process takes place. To handle this issue, the preprocessing module uses an intrinsic relationship resolution strategy. According to this strategy, the instances of all the datasets are sorted to have one-to-one relationships among each other in different datasets based on their ID values. This operation can be done using the sort operation on all the datasets, as shown in Equation (3):

(3) $$DS\prime =\overset{n}{\underset{i=1}{\sum }}intrinRelation\left(ds\prime_{i}\right)$$

In the above equation, the function intrinRelation takes the datasets individually as an argument and sorts the records in ascending order with respect to their IDs. This function writes the sorted dataset back into the same dataset rather than creating new files. After defining the relationship, the instances of the datasets have one-to-one relationships with each other and can be combined. The set of all the sorted datasets is represented by DS′. Here, it is noteworthy that if one dataset labels patient identifiers as “ID” and another as “Patient ID”, then this method is inadequate and we need a synonymous attribute resolution mechanism which is part of future work. Similarly, the problem of one-to-many relationship between the instances of different datasets is also left for future work.

#### 4.2.4. Reduction

Another issue that may create problems during the combination process is the use of different sized datasets. Before applying the combination process, all the datasets should be equal size. To resolve this issue, we propose a reduction technique to make datasets the same size and make them uniform. The motivation behind the reduction strategy is to have data bases with a uniform structure. For example, if all datasets (*1…n*) have the same number of *m* instances then the unified dataset, will have *m* instances. If the datasets have different numbers of instances, then the unified dataset may have a large number of null values resulting in a low quality unified dataset. The reduction technique first checks the number of instances in each dataset and selects the one with the minimum number as the baseline dataset (BD), as shown in Equation (4):

(4) $$BD=ds\left[1:index\;\left(min\left(\sum_{i=1}^{n}Size\left(ds\prime \right)\right)\right)\right]$$

In this equation, first, the size of all the datasets is calculated using the Size function and then min function selects the minimum one as the upper index of the baseline dataset. The lower index of the baseline dataset is set to 1. After finding the size of the baseline dataset, the next step is to reduce all the remaining datasets to the size of this baseline dataset. This process is shown in Equation (5):

(5) $$U=\overset{n}{\underset{i=1}{\sum }}\left[Reduct\;\left(ds\prime_{i},BD\right)\right]$$

In this equation, the Reduct function takes the sorted dataset as an argument and reduces it to the size of the baseline dataset. The reduction method eliminates the records that are not included in the baseline dataset. This process makes all the datasets uniform and ready for fusion. The set of all the uniform datasets is represented by symbol U.

### 4.3. Generating a Unified Dataset

Once the datasets are preprocessed, they need to be combined in the unified dataset using a set of methods. The subsequent sections describe these methods and approaches in detail.

#### 4.3.1. Prioritization Phase

In the case of multiple relevant datasets, there are strong chances that some of the attributes may overlap with each other across multiple datasets. While combining such datasets together, the problem of overlapped attributes occurs. To handle this issue, we propose the idea of defining priority levels *i.e*., an expert-centric priority-based approach. According to this approach, different datasets of a domain have different levels of significance that depend on the credibility of the data source. Therefore, to exploit the credibility feature of the data sources, we let the knowledge engineer assign different priorities to datasets based on feedback from the domain expert. The knowledge engineer may not know the level of significance of the data sources; if the same data is coming from various sources, the domain expert helps the knowledge engineer rank the data sources. The domain expert ranks the datasets according to their importance level. This process is shown in Equation (6):

(6) U= userConfig (U,Priorities)

In the above equation, the userConfig function assigns an appropriate priority value to each dataset*.* The prioritized datasets are written back to the same datasets, rather than creating new copies. The priority values are denoted by $P_{i}$ as shown in Equation (7). The lower priority value shows higher importance and *vice versa*:

(7) $$P=P_{1},\;P_{2},\;\ldots ,\;P_{q}$$

If an expert believes two or more datasets have the same level of significance, then the engineer assigns the same priority values to them. These priorities are important features in the fusion process to handle overlapping attributes.

#### 4.3.2. Defining a Global Unified Data Model

To integrate multiple datasets in a unified way, the first challenge is to define a unified data model and then to log the data. We propose a method which automatically creates the model at run time from all participating datasets. The automatic creation of a model is a unique features of this work and is shown in Equation (8):

(8) M=unifiedDataModel(addAttribute)

where:

(9) $$addAttribute=\{\begin{array}{cc} add\left(a_{j}\right) & \forall \overset{n}{\underset{i=1}{\sum }}\overset{m}{\underset{j=1}{\sum }}\left(u_{i}.a_{j}\right)\notin M\\ ignore & otherwise \end{array}$$

In Equation (8), the unifiedDataModel function adds the attributes of all the datasets, one by one, to the unified data model (M). The process of adding the attributes is represented in the where clause Equation (9) According to this clause, each uniform datasets ($u_{i}\in U$) is sequentially processed for the attribute ($a_{j}$) and added to the model, if it is not already in the model. Otherwise, the attribute is ignored. Repeating this process, attributes from all the datasets are shifted to the model. The overlapped attributes are added only once to the model. In this case the output will be: *M =* {*ID*, *Gender*, *TC*, *TG*, *LDL*, *HDL*, *SGOT*, *SGPT*, *FBS*, *SBP*, *DBP*, *Weight*, *Height*, *Hypoglycemia*, *HbA1c*, *DiabetesType*, *Symptoms*, *Sentiments*, *Activities*}.

#### 4.3.3. Combining Datasets: Logging Data to the Unified Data Model

Once the unified data model is created, the next step is to transfer data to this model from all the datasets. An expert-centric priority-based technique will help at this stage. During the fusion process, the data model (M) is populated with data from two types of attributes of diverse datasets. These are unique and overlapped attributes. Hence, the first step in the fusion process is to identify the lists of all unique and overlapping attributes and then transfer the data to the model. The process of extracting the list of overlapping and unique attributes is shown in Equations (10) and (11):

(10) O=overAttrib(addOAttrib)

where:

(11) $$addOAttrib=\overset{n}{\underset{i=1}{\sum }}\overset{n}{\underset{j=i+1}{\sum }}add(\left[\cap^{\text{​}}\left(u_{i},u_{j}\right)\neq \emptyset \right])$$

The overAttrib function of Equation (9) takes addOAttrib as an argument and adds the overlapping attributes to the list of overlapping attributes (O). The process of finding the overlapped attributes is shown in the where clause (Equation (11)). Here, the intersection of the first uniform dataset, $\;u_{i}\in U$, is taken with all other datasets to find the overlapping attributes. If the result of the intersection is not null, then attribute(s) is/are added to the list O. The process is repeated for all datasets. The output of this process is*: O =* {*ID*, *Gender*, *Activities*}*.*

There may be cases where the attributes among various datasets will be either synonyms or polysemy of each other, e.g., “Gender” or “Sex” or the “Time” that could be the time of recording data or the time that the patient took to finish a test. We have left this case as future work to be solved using polysemous and synonymous attribute resolution mechanisms.

Similarly, Equation (12) shows the process of extracting and adding unique attributes from the list of all uniform datasets (U):

(12) Uniq=addUnique(M\O)

In this equation, the complement of the list of overlapped attributes (O) is taken with the attributes of the unified data model (M) which returns the list of unique attributes (Uniq). This list contains the following attributes: *Uniq =* {*TC*, *TG*, *LDL*, *HDL*, *SGOT*, *SGPT*, *FBS*, *SBP*, *DBP*, *Weight*, *Height*, *Hypoglycemia*, *HbA1c*, *Diabetes Type*, *Symptoms and Sentiments*}.

Once these lists are identified, the next step is to log data into unique and overlapping attributes of the unified data model (M) from all the datasets. For loading data to the unique attributes of the data model, a simple straight forward transfer method is used as shown in Equation (13):

(13) $$M=\overset{n}{\underset{i=1}{\sum }}\overset{m}{\underset{j=1}{\sum }}loadData\left(u_{i}.a_{j}\right)\;\forall \;a_{j}\in Uniq\;\&\;Uniq.\;a_{j}==u_{i}.a_{j}$$

In this equation, the loadData function is used to load data of each unique attribute, $a_{j}\in \;Uniq$, from the diverse datasets $\left(u_{i}\right)$ and shift it into its corresponding attribute in the unified data model (M).

In case of overlapping attributes (O), the “data modeler” *tool* uses our proposed priority-based approach. To shift data from the overlapping attributes, the priority value is checked and the attribute whose dataset has a higher priority value is selected for shifting the value to the unified data model, as shown in Equation (14):

(14) M=loadData(maxPriority)

In Equation (14), the *loadData* function retrieves data from the overlapping attribute whose dataset has higher priority value and transfers it into its corresponding attributes in the unified data model. When the datasets have equal priorities, the simple maximum priority approach is insufficient to handle the overlapping feature problem. To tackle this situation, we propose an additional measure of completeness of the datasets. This measure is calculated in terms of the total number of null or empty values in the datasets. A dataset with a minimum number of null values has a high measure of completeness and is thus considered as a higher priority than the other competitors. This process is shown in Equation (15):

(15) M=loadData(maxCompleteness)

In Equation (15), the loadData function is used to load the data from the dataset whose measure of completeness is maximal.

#### 4.3.4. Exporting Unified Dataset

The unified dataset is important for automatic knowledge acquisition in the form of rules creation. To acquire knowledge using state-of-the-art machine learning algorithms, the dataset must be in a specific file format and structure that is compatible with these algorithms. As our target cloud-based smart *CDSS* is supposed to use machine learning algorithms for knowledge acquisition and reasoning, the unified dataset must be compliant with the formats and structured of these algorithms. For this purpose, implementation of the proposed “data modeler” tool provides export manager as a key module to export the dataset in specific format and structure. This module uses a list of data types (Table 1), supported by the most commonly used data analysis tools, such as *WEKA* [40], *KEEL* [41], *ROSE2* [42], and *RSES* [43].

**Figure 4:**
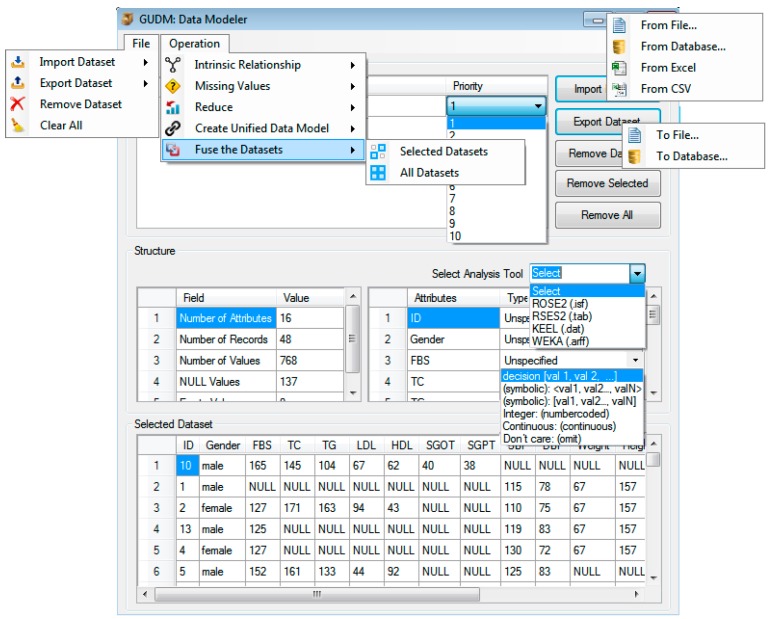
The “data modeler” launch interface with all the supported controls.

**Figure 5:**
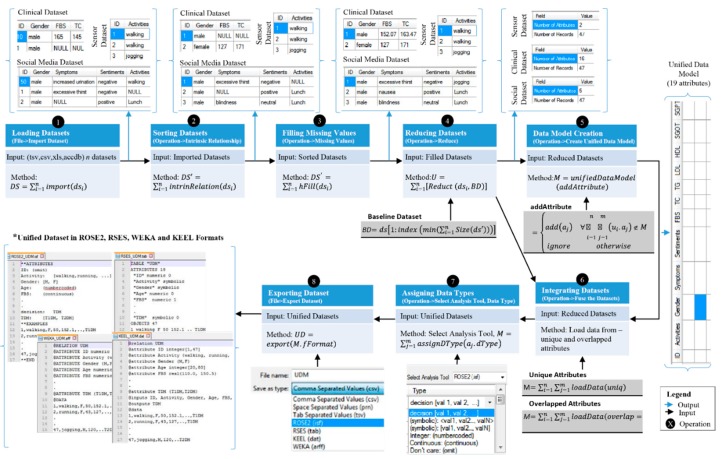
Working of the “data modeler” to create unified dataset in diabetes scenario.

Using these data types, an engineer is pleased to select the appropriate data type for each attribute of the unified dataset (M), as shown in Equation (16):

(16) $$M=\overset{m}{\underset{j=1}{\sum }}assignDType\left(a_{j}.dType\right)$$

where *dType* is the list of all available data types and assignDType is a function used to assign data types to attributes of the unified data model (M). To minimize error chances during assignment of correct data types to attributes and produce high quality unified dataset as output, support for the selection of target data analysis tool is provided by adding a combo box “select analysis tool”, to the “data modeler” It contains the list of all the target tools, as shown in Figure 4*.* Before the export operation starts, target analysis tool is selected from the combo box, which loads data types of that selected tool to the “data modeler” export manager environment. The loaded data types help knowledge engineers and domain experts to assign correct data type to each attribute and minimize errors. Figure 4 and Figure 5 (step 7) shows the process of selecting data analysis tool and assignment of appropriate data types to attributes.

**Table 1:** List of the supported data types in “data modeler” for *ROSE2*, *WEKA*, *KEEL and RSES* data analysis tools.

**(a) ROSE2 (.isf)**		
**Type of Attribute**	**Description**	**Data Type**
Decision	Used for class attribute	decision [val 1, val 2, ...]
Nominal (symbolic)	Used for ordered list	<val1,val2…,valN>
Nominal (symbolic)	Used for unordered list	[val1,val2…,valN]
Integer	Used for positive integer	(numbercoded)
Continuous	Used for continuous numbers	(continuous)
Donʼt care	To be omitted during analysis	(omit)
**(b) WEKA (.arff)**		
**Type of Attribute**	**Description**	**Data Type**
Date	Used for date	date [<date-format>]
Nominal (symbolic)	Used for ordered list	{val1,val2…,valN}
Nominal (symbolic)	Used for unordered list	{val1,val2…,valN}
Integer	Used for positive integer	numeric
Continuous	Used for continuous numbers	numeric
String	Used for string	string
**(c) KEEL (.dat)**		
**Type of Attribute**	**Description**	**Data Type**
Decision	Used for class attribute	outputs
Integer	Used for integers	integer [min, max]
Continuous	Used for continuous	real [min, max]
Nominal	Used for symbolic/strings	nominal {val1, val2, ...}
Inputs	Used for specification of input attributes	outputs
**(d) RSES (.tab)**		
**Type of Attribute**	**Description**	**Data Type**
String	Used for symbolic	symbolic
Symbolic	Used for symbolic	symbolic
Nominal	Used for symbolic	symbolic
Integer	Used for integers	numeric 0
Continuous	Used for continuous numbers. *D* denotes number of digits after decimal point	numeric D

Likewise, the engineer is also given an option to export datasets in multiple file formats, such as *.tab*, *.dat*, *.arff*, *.isf*, *.csv*, *.tsv*, *.prn* and *.accdb.* The process of exporting the dataset is shown in Equation (17):

(17) UD=export(M.fFormat)

Here, the function export is used to assign the required file format (e.g., *.tab*, *.dat*, *.arff*, *.isf*, *.csv*, *.tsv*, *.prn*, .*accdb*) to the unified output dataset and export it to unified dataset, *UD*. Export operation not only export unified dataset in the specified format, but also structure it in the required specific structure of the target analysis tool. Each output unified dataset has its own structure with specific header and data sections. Therefore, when export function is activated, structuring of the unified dataset starts in background in the specified format. Detail of each types of the target output dataset produced as a results of “data modeler” operations, are shown in Figure 5. This is one of the key features of the “data modeler” to let knowledge engineer or domain expert to structure his/her unified dataset correctly without knowing the structure details. The unified dataset is further used for higher level analysis of diabetes which is beyond the scope of this paper.

### 4.4. Unified Dataset: Algorithm for the Creation of Integrated Dataset

The process of unified data modeling and dataset creation is algorithmically presented in Algorithm 1 below.

**Algorithm 1.** Automatic Creation of Unified Dataset**Input**   DS–Set of *n* heterogeneous datasets**Output**   M’–A unified dataset1. [import datasets from the list of *n* available sources]$DS=\overset{n}{\underset{i=1}{\sum }}import\left(ds_{i}\right)$2. [complete missing values, if any]$DS^{\prime }=\overset{n}{\underset{i=1}{\sum }}hFill\left(ds_{i}\right)$3. [make the datasets uniform (*i.e.*, equal size), if not](a) [find intrinsic relationship (*i.e.*, sort the instances w.r.t their IDs), if not]$DS\prime =\overset{n}{\underset{i=1}{\sum }}intrinRelation\left(ds_{i}\right)$(b) [reduce the datasets to equal size]$U=\overset{n}{\underset{i=1}{\sum }}\left[Reduct\;\left(ds_{i},BD\right)\right]$, where $BD=ds\left[1:index\;\left(min\left(\overset{n}{\underset{i=1}{\sum }}Size\left(ds\prime \right)\right)\right)\right]$4. [assign user-defined priorities to each dataset]U= userConfig (U,Priorities)5. [create unified data model]M=unifiedDataModel(addAttribute)6. [log data to the unified dataset](a) [find the list of overlapped attributes]O=overAttrib(addOAttrib)(b) [find the list of unique attributes]Uniq=add(M\O)(c) [log data to the unique attributes of the unified data model]$M=\overset{n}{\underset{i=1}{\sum }}\overset{m}{\underset{j=1}{\sum }}loadData\left(u_{i}.a_{j}\right)\;\forall \;a_{j}\in Uniq\;\&\;Uniq.\;a_{j}=u_{i}.a_{j}$(d) [log data to the overlapped attributes of the unified data model]If (datasets of the overlapped attributes have different priorities)
M=loadData(maxPriority) ElseM=loadData(maxCompleteness)7. [export unified dataset](a) [assign appropriate data type to each attribute]$M=\overset{m}{\underset{j=1}{\sum }}assignDType\left(a_{j}.dType\right)$(b) [export unified dataset in specific format]UD=export(M.fFormat)8. **end**

This algorithm takes n heterogeneous datasets (*i.e*., $ds_{i}$) as input and sequentially passes through each step to produce the intermediate fusion results. Passing through all the mandatory steps of the algorithm generates a unified dataset which can further be used for different types of medical decisions.

### 4.5. Learning and Reasoning from Unified Dataset

The aim of a unified dataset, created from diverse sources, is to use it for data driven knowledge acquisition, where various machine learning algorithms are used to acquire knowledge/rules from the structured data. These rules can best serve the reasoning process for generating various types of prediction services. As the unification process combines datasets from diverse sources, the unified datasets may contain inconsistencies which need to be resolved before the data can be used in any learning or reasoning system/tool. For this reason, in our test case scenario, we adopt rough set theory [46] to learn rules using a *LEM2* algorithm [47] from an automatically generated unified dataset of diabetes cases. We use a rough set exploration system (*RSES*) [48], which implements a suit of rough set algorithms, to create rules from inconsistent data. The unified dataset created using our proposed approach can also be learned by other tools, such as *WEKA*, *KEEL* and *ROSE2* *etc*. Once the rules are created by any of these tools then rule-based reasoning can be applied to predict relevant services. The reasoning part is beyond the scope of this paper and can be considered in future work.

## 5. Working of the Data Modeler: Unified Dataset Creation

To enable the process of unified dataset creation from diverse data sources using the concept of GUDM and a user-centric priority-based approach, the “data modeler” tool, shown in Figure 4, is developed. This tool is an interactive software system with minimal open source support that runs under any 32-bit and 64-bit operating system as a stand-alone application. The open source implementation of the system is helpful for developers in the healthcare community to use, customize and modify the system according to their requirements and needs. This way, the developing community can easily contribute to the system and enhance its functionalities. The core modules of the system are written in *C#* programming language and its *GUI* is designed with an easy-to-use interface with click and menu-driven features to prepare and fuse data. The “data modeler” implementation is module-based and consists of a number of computational modules. Each module is independent of other modules and only requires reading of a dataset to produce a processed dataset as an output. The *GUI* of the “data modeler” has a good overlay between different computational modules of the system. The design and architecture of the system allow the developer and experts to extend it easily by adding new modules to the existing architecture.

The *GUI* of the data modeler has three main controls: *menu*, *frames* and *command buttons*. The menu bar is placed on its standard top left position and has *File* and *Operation* menus. The *File* menu has options for import and export and removing the datasets. The *Operation* menu has options for finding intrinsic relationships, completing missing values, reducing the data, creating a unified data model and fusing datasets.

The frames are labeled as *data sources*, *structure* and *selected datasets*. The *data sources* frame is a container used for holding the imported datasets in their original and intermediate formats. This frame also has a list of priorities that are assigned to the datasets by the domain expert. The second frame, labeled *structure* contains three sections, a combo box labeled “*Select Analysis Tool*”, meta-information and attributes of the dataset. All these details are displayed only for the active (selected/focused) dataset. The combo box is used to select any of the target analysis tool, e.g., *ROSE2*, *RSES*, *KEEL and WEKA*. Meta-information describes meta-statistics including number of attributes, number of records, real values, NULL values and empty spaces contained in the selected dataset. In the attributes section of the structure frame, the list of all attributes of the active dataset is displayed. The final section labeled “*Type*” contains a list of all data types that are used to be assigned to the attributes during the export operation. The third and last frame, labeled “*Selected Dataset*” provides a data view for the selected dataset in data grid format.

## 6. Simulation of “Data Modeler” for Unified Dataset Creation

### 6.1. Case Study: Diabetes Mellitus

To show the significance of the “data modeler” and the proposed model, we consider a scenario where diabetes patients regularly visit a hospital for clinical checkups. Patients are also active on social media regarding their general symptoms and they perform physical activities. Their clinical records are stored in a clinical dataset, social and physical activities information is recorded in social media and in a sensory information dataset, respectively. The clinical dataset is acquired by our lab from a local hospital in anonymized format. This dataset contains 100 patient records, in which 20 patients have type-1 diabetes, 40 have type-2 diabetes mellitus, and 40 have suspicions of diabetes. The attributes of this dataset include: ID, Gender, Total Cholesterol (*TC*), Triglyceride (*TG*), Low-density Lipoprotein (*LDL*), High-density Lipoprotein (*HDL*), Serum Glutamic Oxaloacetic Transaminase (*SGOT*), Serum Glutamic Pyruvic Transaminase (*SGPT*), Fasting blood sugar (*FBS*), Systolic blood pressure (SBP), Diastolic blood pressure (*DBP*), Weight, Height, Hypoglycemia, *HbA1c* and Diabetes type.

Similarly, the social media dataset is obtained from the human aware technology team of the same lab*.* They have developed a Social Media and Interaction Engine (*SMIE*) [49] that consists of three components: one for each Tweet analysis, Trajectory analysis and Email analysis, for collecting patient information from social media profiles. The Tweet analyzer module analyzes the user tweets in order to generate knowledge about user health conditions. They used Alchemy API to extract disease and symptoms from the plain text and perform sentiment analysis. The Trajectory analyzer monitors and tracks the user’s activities through a smartphone embedded GPS sensor. The gender information of the user is extracted from a personal profile of the patient on social media. This dataset contains a total of 50 records with attribute names including ID, Gender, Symptoms, Sentiments, and Activities.

The sensory dataset contains physical activity of patients acquired using embedded sensors of smartphone (*i.e*., accelerometer, audio, *GPS* *etc*.) [50], home environment [51] for recognizing human activities, and video cameras for emotion recognition [52]. This data is processed by individually developed modules attributes (ID and Activities) are used for diabetes patient management in Smart CDSS. The input file format of the “data modeler” tool is a tab-separated text file (.tsv) with the first row as attributes names or MS Access database file (.accdb). Other supported formats includes (.xls) and (.csv).

### 6.2. Working of the Data Modeler

In this section, we describe working of the data modeler for creation of a unified dataset for diabetes scenario. The unification process, for the mentioned clinical, sensory and social media datasets, is described in detail which is depicted in Figure 5. In step 1, all the three datasets are loaded into the “data modeler”, using the import option, either from *file* menu or *command button* control (Figure 4).

The imported datasets have missing values and unequal number of records in unsorted format. The unsorted record issue is resolved, in step 2, using the intrinsic relationship function from the operation menu. The output of this step is the list of datasets with recodes sorted in ascending order. The missing values are represented by NULL keyword in the datasets and resolved in step 3 using missing value filling function. This function can be activated from operation menu. The data modeler fill the missing values in the same datasets or create new datasets and add in data modeler. The option of adding new dataset after filling is asked from the user. Output of step 3 can be seen from Figure 5, where all the filled NULL values are filled with appropriate value. The unequal number of records in clinical dataset are 48, social media dataset are 50 and sensory datasets are 47, which create problems during the fusion process. This is resolved in step 4 (Figure 5) using the reduce function from operation menu. The output of this step is datasets with 47 records, each. This step uses the concept of baseline dataset, dataset with minimum number of records, which is sensory in our case. The fifth step of data modeler is to create unified data model for all the three datasets using the ‘create unified data model’ option, from the operation menu. During the process of data model creation, schema of each dataset is scanned sequentially and the attribute are added to a default schema, named UDM. In our example case, UDM contains 19 attributes out of the total 23 (2 + 5 + 16) for sensory, social media and clinical datasets. Once the model is created, priorities are assigned to each dataset by domain expert from the list of available priorities, as shown in Figure 4. The actual fusion, logging data to UDM, from all the selected datasets is performed in step 6. It is done using the “fuse the dataset” option from the operation menu. In this step, data from the unique and overlapped attributes of the three attributes is logged into UDM. For loading data from overlapped attributes, the priorities-based approach of the proposed study is used. The clinical and social media datasets have gender attributes as overlapped while social media and sensory datasets have activities attribute as overlapped. During the fusion phase, priority 1 is assigned to clinical dataset while 2 and 3 to sensory and social media datasets, respectively. As priority of clinical dataset is higher than social media dataset, therefore gender attribute of the clinical dataset is selected. Similarly, priority of sensory dataset is higher than priority of social media dataset, therefore, activities attribute of sensory dataset is selected. The final unified dataset contains 19 attributes. The unified dataset is of great importance due to its use in further analysis and knowledge acquisition processes. To enable generation of high quality unified datasets as output, the tool is supported with the following options.

- Selection of the target data analysis tool

**Figure 6:**
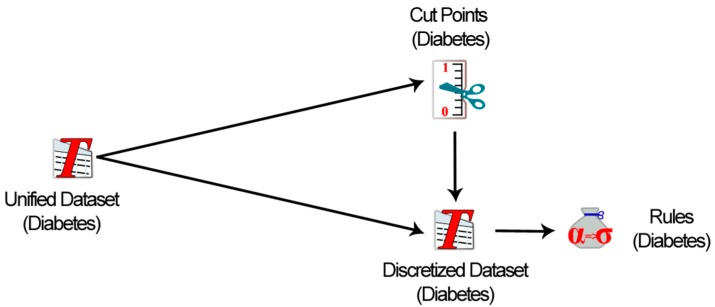
Rules creation process from the unified diabetes dataset using rough set-based algorithm (*LEM2*).

We have provided option for knowledge engineers to first select the target data analysis tool and then generate the unified dataset in specific structure and file format of that tool. Selection of the tool can be done using “select analysis tool” combo box. In Figure 4 and Figure 6 (step 7), the process of selection of the tool is shown. In our example scenario, we select the four mentioned tools, one at a time, to generate unified dataset in four different formats (shown in output of step 8).

- Assignment of the correct data types

To minimize chances of erroneous data types assignment, the tool is supported with the facility of automatically loading only those data types to “type” combo box, which belong to the selected tool. The engineer can easily assign the correct data type to each attribute and thus the errors chances are reduced. In Figure 5, step 7 shows the process of data type assignment for ROSE2 file format.

- Support for specific file structure

As we generate unified dataset for the four mentioned data analysis tools, *i.e*., ROSE2, RSES, WEKA, and KEEL, therefore structures of the files for these tool need to be followed. Though these structures are different, yet they have a common philosophy of having meta-data section, called header, and actual data section. The header section contains information, such as name of the dataset, attributes along with their data types, number of objects/records/examples, specification of inputs and outputs attributes etc. The representation of meta-information is different for all the data analysis tools. The data section of the unified dataset sometimes starts with special tag, such as @data *etc*., and ends with another special tag like @end, but some of the tools have just a new line as the ending of the dataset. The instances within these tags are in one of the formats (.tsv, .csv, or .prn). Understanding the specific constructs of each data analysis tool and structuring dataset accordingly is really a challenging task for domain experts. It wastes a lot of time of experts. To overcome this issue, the data modeler uses internal mapping technique to map each data type against each attribute in the specific format and structure of the dataset and create the final unified dataset in high quality. In Figure 5, output of step 8 shows the unified dataset in ROSE2, RSES, WEKA, and KEEL structures.

- Support for multiple output file formats

The unified dataset can be exported in multiple file formats, such as .csv, .prn, .tsv and MS access database files, ROSE2 (.isf), RSES (.tab), WEKA (.arff), and KEEL (.tab). This gives more options to experts and knowledge engineers to analyses their output dataset in multiple data analysis tools. Once the unified dataset is generated, the rules creation process can be activated. However, this process can be performed outside the “data modeler” environment in other supporting learning tools. In our case, we have used rough set theory-based *LEM2* algorithm implemented in the rough set exploration system (*RSES*) [43]. The rationale behind the use of rough set-based rules creation methodology is its ability to efficiently learn inconsistent data. In this knowledge acquisition process, first we use local discretization algorithm to find cut points and discretize the continuous values of attributes from the unified diabetes dataset and then compute *reducts* which are used in rules creation, as shown in Figure 6. The algorithms used for computation of cut-points, discretization and *reducts* are used from the *RSES* system [43].

To realize the process, a few examples of rules created from the unified diabetes dataset are shown in Table 2.

**Table 2:** A few examples of the rules, created from the unified dataset.

Rule #	Rules
1	If (BMI = [18.5–24.9]) & (Age = (50, Inf)) & (SBP = [120–139]) & (Hba1c = (7.4, Inf)) & (TC = (−Inf, 200)) & (SGPT = [7–56]) = > DiabetesType = 1
2	If (Gender = M) & (SBP = (−Inf, 120)) & (Hba1c = (6.4–7.4]) & (LDL = [100–129]) =>DiabetesType = 2
3	If (BMI = [18.5–24.9]) & (Age = [30–50]) & (SBP = (−Inf, 120)) & (TG = (−Inf, 150)) & (HDL = [40–60]) =>DiabetesType = 2

Legend: symbol “[” or “]” means inclusive, “(” or “)” means exclusive, “–” means to, “Inf” means infinity.

The reasoning and prediction process on the generated rule can be performed for different services which is beyond the scope of this paper.

## 7. Data Modeler Evaluation

The main focus of our proposed study is to create an easy to use software tool for creating high quality unified dataset from diverse datasets so that to be used for reasoning and prediction services. A software that properly implements the most desirable features of unification process is more useful regarding time aspect, in contrary to the complex systems take consume more time in producing the same results. Hence, we have considered time factor is our evaluation criteria. We have evaluated “data modeler” tool for creating six unified datasets by three domain experts and three knowledge engineers. Domain experts were experienced with the individual diverse datasets and knowledge engineers were medium and expert level professionals in the area of data unification. Total fifteen diverse datasets with different complexity levels were grouped in six different sets, each of which was assigned to a single participant of the evaluation team. The datasets were locally generated from the biomedical data. The characteristics of all the participants and the datasets are provided in Table 3.

Before starting evaluation, we have provided the group of datasets to the evaluators and delivered training on the use of “data modeler” tool to create unified dataset using the steps described in Section 6.2. The evaluators were also instructed to use the traditional MS Excel program to perform the same operations. For experiments, each participant were asked to create the unified dataset for his/her assigned set of datasets both in “data modeler” tool and manually in MS Excel as per our instructions. We have recorded the time spent by each participant for completing the task of unified dataset creation using both the tools. The time taken by each evaluator is shown in Table 4.

**Figure 7:**
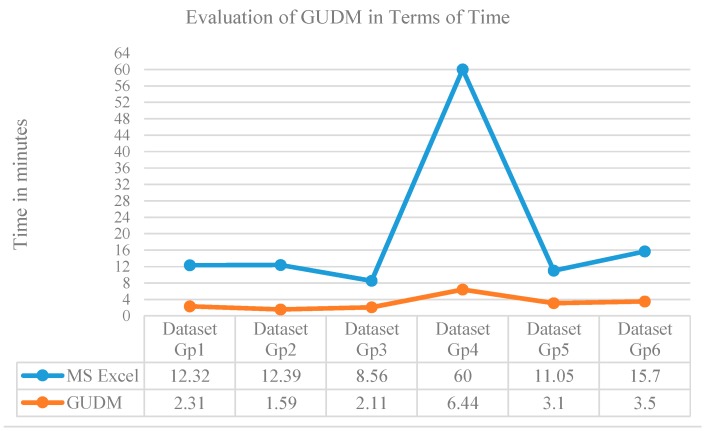
Time comparison of the proposed data modeler with traditional MS Excel program.

We have observed from our experiments that the proposed data modeler enhance average performance of both the domain expert and knowledge engineer by 84.1 percent *i.e*., saves 84.1 percent time of them. Evaluating the performance separately for expert and knowledge engineer, the tool saves 81.9% of expert time while 84.9% of the knowledge engineer. The time comparison of the proposed toll with the MS Excel program, where the datasets are manually combined to a unified dataset, is shown in Figure 7.

**Table 3:** Characteristics of the test datasets used for evaluating “data modeler” to create unified dataset.

Dataset Group	Dataset	No. of Attributes	No. Records	No. of Overlapping Attributes	%Age of Missing Values	Uniformity (Same Size)	Intrinsic Relationship (Sorted Records)	Evaluator
Gp1	testDataset1	4	5	2	5	yes	yes	Domain expert1
	testDataset2	3	5	2	6.5			
Gp2	testDataset3	9	10	3	30	no	no	Domain expert2
	testDataset4	4	10	3	10			
Gp3	testDataset5	3	5	2	13.3	yes	no	Domain expert3
	testDataset6	4	5	2	10			
Gp4	testDataset7	16	48	2	15	no	no	Knowledge engineer1
	testDataset8	2	47	2	0			
	testDataset9	5	49	3	8.5			
Gp5	testDataset10	6	20	2	4.5	no	no	Knowledge engineer2
	testDataset11	6	20	2	6.5			
	testDataset12	6	20	2	2			
Gp6	testDataset13	7	20	3	3	no	no	Knowledge engineer2
	testDataset14	5	29	2	5			
	testDataset15	9	35	1	10			

**Table 4:** Evaluation results of the data modeler in terms of time taken during unified dataset creation.

Datasets Group	Unified Dataset	Evaluator	Unified Dataset Creation Time	
			Traditional Tool (MS Excel)	GUDM (Data Modeler)
Gp1	UD1	Domain expert1	12.32	2.31
Gp2	UD2	Domain expert2	12.39	1.59
Gp3	UD3	Domain expert3	8.56	2.11
Gp4	UD4	Knowledge engineer1	60	6.44
Gp5	UD5	Knowledge engineer2	11.05	3.10
Gp6	UD6	Knowledge engineer2	15.7	3.5
Average time taken			20.00333	3.175
Average reduction of time			16.82833	
Percent reduction of time			84.12765	

## 8. Discussion of Significance, Challenges and Limitations of the Work

Data fusion and integration applications are gradually being introduced in medical practice. The time of a knowledge engineer is precious. We have proposed a system that aims to reduce the amount of time an engineer spends gathering information from multiple datasets and integrating them. To automatically integrate multiple datasets into a unified dataset, this paper has implemented an expert centric priority-based approach. The purpose of the unified dataset is to use machine learning approaches to predict different diseases (*i.e*., diabetes in this case). Though the implemented approach combines data only from non-imaging data sources, it can solve the problem of overlapping features in the datasets. We expect that the fusion of heterogeneous data (social media and sensor activities) with the clinical record will further improve prediction accuracy. It will provide a more sensitive measure of all significant parameters of diabetes disease. The proposed framework has the potential to be extended for further development of other modalities for unified analysis. The framework of the “data modeler” tool has the potential to assist in medical predictions while integrating clinical records with other supporting information.

The implementation of our proposed priority-based approach and development of a data fusion system is accompanied by a number of challenging issues. Some of the hard challenges we faced were identification of overlapping attributes, breaking the tie case of priorities of overlapping attributes and presenting the output (dataset) in multiple file formats. To identify overlapping features, we have used the idea of string matching at the attribute names-level. Similarly, when the datasets have the same level of priorities, the tie is broken using the idea of dataset completeness. The dataset with the fewest missing values has a high level of completeness and is therefore considered more prior. Furthermore, to increase the usability of the unified dataset, multiple file formats with all possible data types can be used. This makes it easy for a knowledge engineer to obtain the unified datasets in his/her required format.

Shortcomings of the proposed approach include lack of a dynamic priority assignment, neglecting priorities at the attribute-level and focusing only on a syntactic string matching technique. Moreover, support of tab-separated text files as input limits the use of the system. Other similar limitations that exist are resolving polysemous and synonymous attributes among different datasets during the process of finding overlapping attributes, conflict resolution between the columns of different datasets, fusion when the datasets have a one-to-many relationship, focus on in-memory implementation and the use of a primary key concept during fusion.

## 9. Conclusions

This article describes the problem of fusing multiple heterogeneous datasets into a unified dataset for different types of high-level analysis, knowledge acquisition and reasoning. To accomplish this, an expert-centric priority-based approach has been proposed and implemented as the “data modeler” tool. This application has an extensible framework with an easy to use *GUI* that allows knowledge engineers to import multiple heterogeneous datasets using its import manager and combines them together to obtain the unified dataset. This system supports a number of features such as finding mutual relationships, reducing the datasets, supplying the missing values, defining a unified data model and fusing the data together. The work of the data modeler is illustrated with the help of a case study with three types of heterogeneous datasets containing diabetes patients’ information. From the case study and simulation process, we believe that “data modeler” can be best used to solve a number of problems commonly encountered by biomedical engineers during the data preparation and fusion process. In the example scenario, it is possible to understand the potential of GUDM. The current version of this tool (version 1.0) can be downloaded from the Sourceforge open source page [11].

In the future, we intend to identify methods of integrating more diverse data sources such as imaging and aligning their output for people with different types of diseases. We plan to define a dynamic strategy to automatically measure the importance of the datasets and their attributes and then use it to handle overlapping attributes. We will consider the design and development of a more advanced and powerful editor to tackle multiple file formats to extend the system usability. Another challenging future problem is resolving polysemous and synonymous attributes among different datasets. Solving this problem would identify overlapping attributes among various datasets, resolve conflicts between the columns of different datasets, and enable fusion for the datasets that have a one-to-many relationship. It will also resolve the problem of large datasets that cannot be handled using in-memory implementation.
